# Mode Switch of Ca^2 +^ Oscillation-Mediated Uterine Peristalsis and Associated Embryo Implantation Impairments in Mouse Adenomyosis

**DOI:** 10.3389/fphys.2021.744745

**Published:** 2021-11-04

**Authors:** Mingzi Qu, Ping Lu, Karl Bellve, Lawrence M. Lifshitz, Ronghua ZhuGe

**Affiliations:** ^1^Department of Microbiology and Physiological Systems, University of Massachusetts Medical School, Worcester, MA, United States; ^2^Program in Molecular Medicine, University of Massachusetts Medical School, Worcester, MA, United States

**Keywords:** myometrium, embryo implantation, adenomyosis, Ca^2+^ oscillations, uterine peristalsis

## Abstract

Adenomyosis is a debilitating gynecological disease of the uterus with no medicinal cure. The tissue injury and repair hypothesis for adenomyosis suggests that uterine hyperperistalsis or dysperistalsis plays a pivotal role in establishing adenomyotic lesions. However, specific impairments in uterine peristalsis and the underlying cellular signals for these changes in adenomyosis remain elusive. Here, we report a precision-cut uterine slice preparation that preserves *in vivo* uterine architecture and generates peristalsis similar to that seen in the whole uterus. We found that uterine peristalsis in neonatal mice at day 14 and adult mice at day 55 presents as bursts with multiple peaks induced by intracellular Ca^2+^ oscillations. Using a mouse model of adenomyosis induced by tamoxifen, a selective estrogen receptor modulator, we discovered that uterine peristalsis and Ca^2+^ oscillations from adenomyotic uteri on days 14 and 55 become spikes (single peaks) with smaller amplitudes. The peak frequency of Ca^2+^ oscillations or peristalsis does not show a difference between control and adenomyotic mice. However, both the estimated force generated by uterine peristalsis and the total Ca^2+^ raised by Ca^2+^ oscillations are smaller in uteri from adenomyotic mice. Uteri from adenomyotic mice on day 14, but not on day 55, exhibit hyperresponsiveness to oxytocin. Embryo implantations are decreased in adenomyotic adult mice. Our results reveal a mode switch from bursts to spikes (rather than an increased peak frequency) of uterine Ca^2+^ oscillations and peristalsis and concurrent hyperresponsiveness to oxytocin in the neonatal stage are two characteristics of adenomyosis. These characteristics may contribute to embryo implantation impairments and decreased fertility in adenomyosis.

## Introduction

Adenomyosis is a uterine disorder characterized by the presence of sex hormone-sensitive endometrial glands and stromal cells within the myometrium. Incidence of adenomyosis, often not diagnosed, ranges from 10 to 60% of premenopausal women (Samimi et al., 2020; Upson and Missmer, 2020; Yu et al., 2020). Adenomyosis is often associated with menorrhagia (heavy menstrual bleeding), dysmenorrhea (painful menstrual periods), chronic pelvic pain, dyspareunia (pain with intercourse) and infertility (Vannuccini et al., 2017; Vercellini et al., 2019; Bourdon et al., 2020; Garcia-Velasco, 2020; Yoldemir, 2020; Zhai et al., 2020). These symptoms affect patients’ life and may lead to hysterectomy. Other than via a hysterectomy, adenomyosis cannot be cured (Benetti-Pinto et al., 2019; Cope et al., 2020). Therefore, adenomyosis imposes significant health and socioeconomic burdens on modern society. Current therapies are ineffective in most patients, highlighting the need for novel therapies.

The etiology and pathogenesis of adenomyosis remain elusive. One hypothesis proposes that metaplasia *de novo* of displaced embryonic pluripotent Mullerian remnants or differentiation of adult endometrial epithelial and mesenchymal stem cells in the myometrium is the origin of adenomyosis (Koike et al., 2013; Stratopoulou et al., 2020; Zhai et al., 2020), like in endometriosis (Hufnagel et al., 2015). Another hypothesis suggests that adenomyosis results from the invagination of the endometrium’s basal layer into adjacent myometrium as the result of physiological trauma or physical trauma at the endometrial-myometrial interface (Mehasseb et al., 2010b; Leyendecker and Wildt, 2011). The physiological trauma may be induced by chronic intensive uterine peristalsis while the physical trauma may cause the abnormal uterine peristalsis. The potential role of uterine peristalsis in the pathogenesis of adenomyosis drives the need to understand uterine peristalsis.

Uterine peristalsis is the coordinated spontaneous myometrial contraction and relaxation occurring in the non-pregnant uterus (Eytan and Elad, 1999; van Gestel et al., 2003; Bulletti et al., 2004; Mehasseb et al., 2010b). The myometrium, residing between the endometrium and serosa, is comprised of an inner circular layer and an outer longitudinal layer of uterine smooth muscle cells (USMCs), and a middle layer of supporting connective and vascular tissue. Magnetic resonance imaging (MRI) and non-invasive transvaginal ultrasound (TVUS) reveal that solely the inner circular layer of smooth muscle generates peristalsis, propagating from the fundus to the cervix (or in reverse) at distinct frequencies throughout menstrual cycles (van Gestel et al., 2003; Kissler et al., 2004; Lesny and Killick, 2004; Fanchin and Ayoubi, 2009). Physiologically, this peristalsis plays a fundamental role in normal reproduction by removing menstrual debris, transporting sperm in the female genital tract for fertilization, and transporting the embryo to a proper implantation site on the uterine wall (Crane and Martin, 1991; Ijland et al., 1996; Kunz et al., 1996, 2006; Leyendecker et al., 1996; Kissler et al., 2004; Ye et al., 2005; Bulletti and de Ziegler, 2006; Zervomanolakis et al., 2007; Chen et al., 2011; Zhu et al., 2014). However, iatrogenic procedures such as dilation and curettage surgery or chronic intensive uterine peristalsis throughout a woman’s reproductive lifetime (Curtis et al., 2002; Ibrahim et al., 2015; Hao et al., 2020) may injure the uterine wall, especially in the endometrial–myometrial interface near the fundo-cornual raphe in humans (Leyendecker and Wildt, 2011; Zhai et al., 2020). The damaged uterine tissue then increases local production of estrogen, which stimulates uterine peristalsis via the estrogen receptor alpha (ERα) and oxytocin receptor, leading to further damage of the uterine wall (Orazov et al., 2020). Thus, a vicious positive feedback loop is set up in which chronic uterine peristalsis promotes repeated cycles of injury to the endometrial–myometrial interface, augmenting invagination of the endometrial basalis into the myometrium and leading to adenomyotic lesions. Direct intrauterine pressure measurements reveal that uterine peristalsis can increase uterine cavity pressure, implying that the uterine wall is stressed and strained during the contraction (Martinez-Gaudio et al., 1973; de Vries et al., 1990; Eytan et al., 2001; Ueda et al., 2020). A 2D computer model establishes that uterine peristalsis can exert stress maximally in the endometrial–myometrial interface (Shaked et al., 2015). Thus, uterine peristalsis can be a contributing cause in the evolution of adenomyosis.

Although uterine peristalsis has been identified as a notable cause for adenomyosis’s pathogenesis, how uterine peristalsis may vary between healthy and adenomyotic uteri is not understood, nor are the cellular signals underlying uterine peristalsis. This hinders the translation of the knowledge on uterine peristalsis to adenomyosis management. The poor understanding of uterine peristalsis in adenomyosis is in part owing to the limitations in techniques being used to study uterine peristalsis. MRI and TVUS can detect uterine peristalsis but cannot measure the amplitude of uterine contractions (i.e., its strength or force). Also, TVUS measurement is operator-dependent and is rather subjective. Hysterosalpingo-radionuclide scintigraphy (HSSG) uses a gamma camera to record the migration of radioactive tracers in a suspension injected into the uterine cavity, so it can give a meaningful measurement of uterine peristalsis’ direction and its interpretation is more objective than MRI and TVUS (Fujiwara et al., 2004; Campo et al., 2012; Gorgulu and Okcu, 2020). But this method suffers from its inability to evaluate the amplitude and frequency of uterine peristalsis (Nakai et al., 2004; Kuijsters et al., 2017; Gorgulu and Okcu, 2020). Before MRI, TVUS and HSSD, intrauterine pressure was employed to assess uterine peristalsis (Martinez-Gaudio et al., 1973). Using multiple pressure transducers permits this method to record pressure in different regions of the uterus simultaneously. However, this method is invasive and gives limited spatial information on uterine peristalsis. Another concern with this method is that the pressure probes may induce or alter uterine peristalsis via a short loop reflex. Finally, *ex vivo* isolated uterine strips have been often used to measure uterine contractions via an organ bath (Massenavette et al., 2017; Arrowsmith et al., 2018; Malik et al., 2021). This method does not provide spatial information on uterine peristalsis, nor can it measure wave-like contractions that naturally occur in the intact uterus. This configuration may alter uterine contractile characteristics because of the extrinsic load applied to the strips. Thus, it is necessary to develop new methodologies to study uterine peristalsis to advance understanding of this essential uterine activity in normal reproduction and adenomyosis.

Tissue slices preserve the *in vivo* original cell architecture and have been used to study physiological functions in various organs including pregnant human uteri (Bergner and Sanderson, 2002; Burdyga et al., 2009; Bru-Mercier et al., 2012; Peppiatt-Wildman et al., 2012; Sanchez-Cardenas et al., 2012; Lu et al., 2021a). In conjunction with imaging approaches, tissue slices offer opportunities to reveal cellular signals with high spatiotemporal detail. Considering that uterine peristalsis results largely from the coordinated contraction of circular myometrial cells (van Gestel et al., 2003; Kissler et al., 2004; Lesny and Killick, 2004; Ye et al., 2005; Fanchin and Ayoubi, 2009; Chen et al., 2011), we developed a protocol to cut mouse uteri transversely to generate precision-cut uterine slices. With imaging, the circular USMCs in precision-cut uterine slices were revealed to contract and relax like *in vivo* uterine peristalsis. By loading the tissue with Ca^2+^ indicators or using the tissue from transgenic mice expressing fluorescent calcium indicators, these slices’ contraction and relaxation were correlated with Ca^2+^ oscillations in a one-to-one fashion. Applying this preparation and technique to a well-developed tamoxifen-induced mouse model of adenomyosis, we further discovered that uterine peristalsis and Ca^2+^ oscillations were changed from bursts with multiple peaks in normal mice to largely spikes with a single peak in adenomyosis (in both neonatal and adult stages). When the individual peaks in bursts were counted as separate events, the frequencies of Ca^2+^ oscillation and peristalsis were not different between control mice and adenomyotic mice. The estimated total force generated by uterine peristalsis and the total Ca^2+^ raised during Ca^2+^ oscillations were greater in control slices than in adenomyotic slices. The neuronal secretory factor oxytocin (which dose-dependently increases uterine peristalsis) was more effective in neonatal adenomyotic mice than in control mice. However, this difference disappeared in adult adenomyotic mice. Finally, adenomyotic mice have decreased fertility because of unsuccessful embryo implantation. This study reveals that a mode switch from bursts to spikes, rather than an increase in the frequency, of uterine Ca^2+^ oscillations and peristalsis is a feature of adenomyosis. The hyper-responsiveness to oxytocin in neonatal mice but not in adult mice indicates that uterine Ca^2+^ oscillations and peristalsis can present as a causal element of adenomyosis. These changes in Ca^2+^ oscillations and peristalsis may contribute to reproduction impairments in adenomyosis.

## Materials and Methods

### Mice

All experimental protocols for animal research were approved by the Institutional Animal Care and Use Committees at the University of Massachusetts Medical School (UMMS) (PROTO202000131) following the National Research Council Publication Guide for the Care and Use of Laboratory Animals and NIH Guide for the Care and Use of Laboratory Animal. Mice were maintained under a standard 12-h light/dark cycle (lights on at 7:00 AM) with food and water *ad libitum* (room temperature 22 ± 2°C). CD-1 and C57BL/6J mice were purchased from the Jackson Laboratories and bred in the UMMS animal care facility. Red-shifted genetically encoded calcium indicators report lines using R-CaMP1.07 were generated by Michael Kotlikoff at Cornell University (Ohkura et al., 2012) (CHROMus line acta2-R-CaMP1.07; Jackson stock #028345). αSMA-hrGFP mice were provided by Alan Fine at Boston University (Paez-Cortez et al., 2013).

### Tamoxifen Treatment

To induce adenomyosis in mice, we used a tamoxifen protocol as developed by Parrott et al. (2001). The stomach of neonatal mice when filled with milk is clearly white and readily recognizable. In our preliminary study, we found it was straight-forward to deliver 20 μl solution via injection to the stomach as confirmed with Chicago Blue dye. The stomachs of female neonatal CD-1 mice were injected on days 1 to 5 after birth (day of birth = day 0) with 1 mg/kg tamoxifen suspended in a peanut oil/lecithin/condensed milk mixture (2:0.2:3 v/v). Control mice received vehicle only. Mice on day 14 (i.e., postnatal day 14, PND14) or PND55 ± 3 in estrus were humanely euthanized using CO_2_ and uteri were removed for histological examination or functional tests. These two stages were chosen for investigation because by PND14, the uterus’ basic adult configuration is established (Hu et al., 2004a, b) and by PND55, mice mature sexually. Moreover, investigating two developmental stages of mice after tamoxifen treatment allowed us to explore the natural histology and the root cause of adenomyosis pathogenesis.

### Timed Mating

Sexually mature female mice (7 weeks of age) were mated to fertile males, and copulation was confirmed by observing vaginal plugs the following morning. The morning when the plug was observed was designated as 1 dpc (day post copulation).

### Implantation Site Visualization

Implantation sites were visualized in 5 dpc pregnant mice by tail vein injection of 200 μL of 1% Chicago Blue dye; mice were humanely euthanized 20 min after injection. Dissected uteri were photographed in white light, and blue bands were counted as implantation sites.

### Precision-Cut Uterine Slice Preparation

Previous *in vivo* studies show that uterine peristalsis results from circular muscle contraction and relaxation (Eytan et al., 2001; Bulletti et al., 2004; Brosens et al., 2010). Therefore, we cut the uterus perpendicularly to its long axis such that uterine slices were able to contract and relax in the same direction as uterine peristalsis *in vivo*. Mice were humanely euthanized by carbon dioxide, followed by cervical dislocation. The uterus was removed and transferred to ice-cold Hanks’ balanced salt solution (HBSS, Cat#1387, Sigma Aldrich), supplemented with 20 mM HEPES buffer and adjusted to pH 7.4 (named as sHBSS). After removing adipose and connective tissue, the half of the uterus near the ovarian side was cut and embedded in 5% low melt agarose gel, as was previously done in other types of smooth muscle tissue (Bergner and Sanderson, 2002; Lu et al., 2021a). After the agarose was gelled at 4°C, the specimen was loaded into a compresstome vibratome (VF-300; Precisionary Instruments) and sectioned into 250 μm thick slices. The slices were collected in a 24-well plate with cold sHBSS buffer and examined under an optical microscope. Those slices with intact circular smooth muscle rings were selected for subsequent experiments.

### Uterine Contraction and Ca^2+^ Signal Measurements

Uterine slices were placed on cover glass (45 mm × 50 mm) and held in position with a nylon mesh with a hole cut in the middle. Another smaller cover glass was placed on top of the nylon mesh and sealed on both sides with silicone grease to create a perfusion chamber. The chamber was mounted on an inverted microscope (IX71, Olympus) with a 2× or 4× objective (Nikon, Tokyo, Japan). The perfusion flow rate was ∼700 μl/min driven by a gravity perfusion system via a VC-6 six channel valve controller (Warner Instruments Corp.). All experiments were conducted in a custom-made Plexiglas chamber complete with a custom-built objective heater and a thermal controller to regulate the temperature at ∼33°C.

For uterine contraction measurements, phase-contrast images were collected at a rate of 1 Hz with a CCD camera controlled by the μManager image acquisition software in the time-lapse mode. The contraction and relaxation of the uterus, after a 30 min incubation, were represented by the change of the whole uterine slice area, as described previously (Tan and Sanderson, 2014; Lu et al., 2021a). Briefly, the whole slice’s area was measured by adjusting the gray level threshold in the 1st frame of the image series (ImageJ > Image > Adjust > threshold) such that the entire area enclosed by the uterus was a single particle (i.e., object), and then counting the pixels in that particle (ImageJ > analyze > analyze particles) in ImageJ^1^ (NIH). Changes in the uterine area were calculated as (Area_*t*_ − Area_0_)/Area_0_^∗^100, where Area_0_ is the slice area when the uterus is at relaxation and Area_*t*_ is the slice area at a time point. As uterine peristalsis and Ca^2+^ signals (see below) occurred as bursts (i.e., comprised of multiple peaks) or spikes (i.e., single peaks), the frequency of events was expressed as either the number of bursts plus the number of isolated spikes per second or the number peaks per second. Uterine peristalsis frequency were determined using the local maximum method of the Peak Analyzer in Origin 2020b. The values of local points and amplitude height% were finely tuned such that more than 95% of peaks were found. The missing peaks or falsely selected peaks were manually added or deleted by confirmation via visual inspection of the original images. When events were determined based on bursts or spikes, their full width at half maximum (FWHM) values were calculated. Please note that the contraction spikes in this study may not be correlated one to one with underlying electrical signals, i.e., action potentials, or electrical spikes.

For the uterine Ca^2+^ signal measurement, slices were loaded with sHBSS with 20 μM Cal-520 AM (AAT Bioquest, Inc., Sunnyvale, CA, United States) and 0.06% Pluronic F-127 in the dark at 33°C for 50 min, followed by sHBSS alone for an additional 30 min at room temperature. Uterine slices were then mounted in the perfusion chamber (described above), and fluorescence images were obtained with μManager in a custom-built wide-field digital imaging system (Navaroli et al., 2012). The camera was interfaced to an IX71, Olympus inverted microscope, and images were recorded with a 2× or 4× objective at a speed of 1 Hz. The 488 nm line of an argon-ion laser provided fluorescence excitation, with a shutter to control exposure duration; emission of the Ca^2+^ indicator was monitored at wavelengths >500 nm. The uterine slices’ fluorescence intensity was defined as the average intensity value of the circular smooth muscle region. This region was manually outlined at key frames when the uterine slice’s contraction or relaxation initiated, and outlines on intermediate frames were created by the ImageJ interpolate ROIs function which assumes a linear progression between the key frames. A linear bleach correction with a slope calculated using a period without any Ca^2+^ events was applied using the ImageJ Stacks-T function plugin. Changes in fluorescence intensity were expressed as (*F*_*t*_ − *F*_0_)/*F*_0_^∗^100, i.e., Δ*F*/*F*_0_ × 100, where *F*_*t*_ was the fluorescence intensity at a particular time, and *F*_0_ was the mean fluorescence at rest. The frequency and FWHM of Ca^2+^ oscillations were calculated as described above in Origin 2020b.

### Immunohistochemistry

Protein distribution was assessed by immunohistochemistry as described (Lu et al., 2021a). Uterine cryosections were 8 μm, and antibodies were mouse monoclonal antibody to MYH11 (ab683 clone 1G12, 1:200; Abcam) and Alexa Fluor 488-conjugated goat anti-mouse IgG (Cell Signaling Technology, dilution 1:500). Immunoreactivity was evaluated using a Leica TCS SP8 confocal laser scanning microscope system (Leica Microsystems Inc., Buffalo Grove, IL, United States).

### Hematoxylin–Eosin Staining

Uterine histology was assessed with hematoxylin–eosin stain according to the manufacturer’s instructions (ab245880, Abcam). The stainings were evaluated using a Zeiss Axioplan 2 microscope equipped with a BFLY-U3-23S6C-C USB 3.0 Blackfly camera (Edmund Optics, Barrington, NJ, United States).

### Reverse-Transcription PCR and Quantitative Real-Time PCR

Uteri from mice were isolated and cleaned by removing connective tissues. Total cellular RNAs from specimens were isolated, cleaned and reverse-transcribed as described (Lu et al., 2021b).

qRT-PCRs were carried out to determine mRNA levels of genes with iTaq^TM^ Universal SYBR^®^ Green Supermix (Cat #172-5121; Bio-rad) as described (Lu et al., 2021b). Each gene’s expression level was calculated using the 2^–Δ*Ct*^ method and normalized against the housekeeping gene β-actin. Those with *C*_*t*_ > 35 were considered low precision and counted as no expression as a 35-cycle PCR reaction should amplify even a single transcript to a measurable level. All the primers are listed in Table 1.

**TABLE 1:** Primer sequences for qPCR.

Name	Sequence
αSMA-F	TGTGCTGGACTCTGGAGATGGT
αSMA-R	CGTCAGGCAGTTCGTAGCTCTT
β-Catenin-F	CATGGAGGAGATAGTAGAAGGGT
β-Catenin-R	CCTCAGACATTCGGAATAGGAC
Col1a1-F	ATCACTGCAAGAACAGCGTAGC
Col1a1-R	AATGTCCAAGGGAGCCACATC
Col3a1-F	GGCCAGTGGCAATGTAAAGAAG
Col3a1-R	CCCAATGTCATAGGGTGCGATAT
E-Cadherin-F	CGACCGGAAGTGACTCGAAATG
E-Cadherin-R	GCCCTCGTAATCGAACACCAAC
Esr1-F	CAGACACTTTGATCCACCTGATG
Esr1-R	GAGATGCTCCATGCCTTTGTTAC
Esr2-F	TGCCCTGGTCTGGGTGATTTCG
Esr2-R	AGGTTCTGGGAGCCCTCTTTGC
Gper-F	TTAACAGAGCAGCGATCTGGACC
Gper-R	CAGGCATTTGTGAGGCAGGAAG
Kras-F	CAATGAGGGACCAGTACATGAG
Kras-R	GTATAGAAGGCATCGTCAACAC
Oxtr-F	AATCCGCACAGTGAAGATGACC
Oxtr-R	ATGGCAATGATGAAGGCAGAAG
Tgfb1-F	TTAGGAAGGACCTGGGTTGGAAG
Tgfb1-R	TAGTAGACGATGGGCAGTGGCTC
Tgfbr1-F	GCAATGGGCTTAGTGTTCTGGG
Tgfbr1-R	GCCTTCCTGTTGGCTGAGTTGT
β-Actin-F	AGGCCAACCGTGAAAAGAT
β-Actin-R	AGAGCATAGCCCTCGTAGA

### Statistical Analysis

Data are presented as means ± SE of *N* mice unless otherwise stated. Statistical analyses of characteristic differences of normal and adenomyotic mice were carried with unpaired Student’s *t*-test in the GraphPad Prism 8. The dose responses to oxytocin were analyzed using a two-way ANOVA, and implantation sites were tested with a Chi-Square statistic. The significance levels using mouse number were showed as follows: N.S., no statistical significance (*P* > 0.05), ^∗^*P* < 0.05, ^∗∗^*P* < 0.01, ^∗∗∗^*P* < 0.001, ^****^P < 0.0001.

## Results

### Novel Uterine Slices for Studying Ca^2+^ Signals and Uterine Peristalsis

**FIGURE 1:**
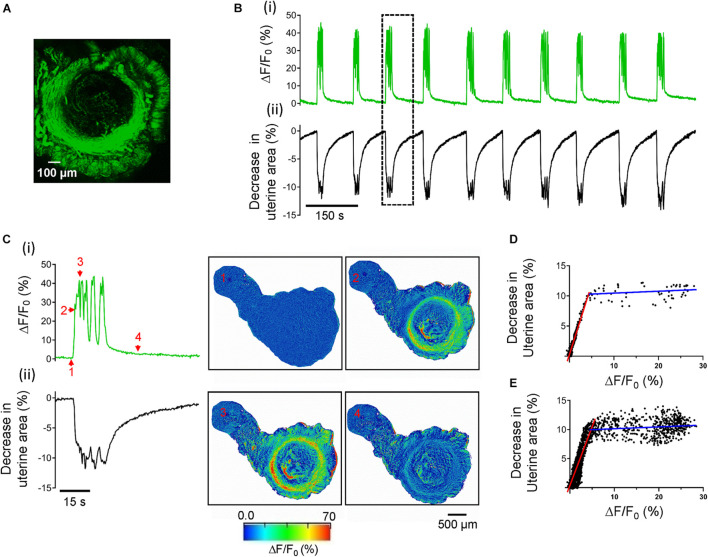
Ca^2+^ oscillations cause uterine peristalsis in precision-cut mouse uterine slices. **(A)** A uterine slice from αSMA-hrGFP mice shows GFP positive signals only in circular and longitudinal myometrial layers. **(B)** Temporal relationship between Ca^2+^ oscillations and uterine peristalsis in a precision-cut mouse uterine slice. Δ*F*/*F*_0_ (%) **[B(i)]**, a proxy for [Ca^2+^]_*i*_, was the averaged value of the entire circular myometrium, while changes in the uterine area **[B(ii)]** were calculated using the whole slice. The event within the dotted line box is shown on an expanded scale in panel **(C)**. **[C(i)]** The evolution of a burst of Ca^2+^ oscillations; **[C(ii)]** The evolution of associated uterine peristalsis. The images on the right display temporal changes in the uterine area and the fluorescence as Δ*F*/*F*_0_ (%), and numbers in the images correspond to those marked near the green line in panel **C(i)**. Note that red signals near the edge of the slices in this figure (and in Figures 6C, 8C) are the artifacts due to the slice contraction and relaxation. **(D)** Relationship between [Ca^2+^]_*i*_ and associated decreases in the uterine area for the event in panel **(C)**. **(E)** Relationship between [Ca^2+^]_*i*_ and associated decreases in the uterine area from all the events in panel **(B)**. Red and blue lines in panel **(D,E)** are the fitting results of two ranges of Δ*F*/*F*_0_.

We used a vibratome to cut the uterine transversely into 250 μm thickness slices to study uterine peristalsis. We first confirmed myometrial structure utilizing a line of the transgenic mouse in which hrGFP is expressed under the control of the α-smooth muscle actin (αSMA) gene promoter (Paez-Cortez et al., 2013). Figure 1A shows that the precision-cut uterine slice has two layers of GFP positive SMCs: the inner circular one as a ring and the outer longitudinal one as bundles. These structural arrangements in smooth muscle cells indicate uterine slices keep their *in situ* cell architecture.

We then assessed the contractile properties of the uterine slices. After a 30 min perfusion with Ca^2+^ buffer at 33°C, uterine slices developed stable spontaneous rhythmic phasic contractions and relaxations (Figure 1B) that persist for hours (data not shown). These activities changed the uterine slices’ size, i.e., the uterine slices underwent radial and isotonic contraction just as they would *in vivo*. As revealed in the Kymographs in Supplementary Figure 1, each rhythmic contraction and relaxation induced the same spatial change in the uterine slice. To quantify the contractions and relaxations, we measured the entire uterine slice’s size over time using ImageJ software’s particle detection function. Figure 1B(ii) shows the time course of changes in the size of a uterine slice. Given the mouse uterus is a tube structure, and coordinated uterine circular smooth muscle contractions traveling through the tube constitute peristalsis (van Gestel et al., 2003; Kissler et al., 2004; Lesny and Killick, 2004; Fanchin and Ayoubi, 2009; Moliner et al., 2021), it is arguable that rhythmic contractions and relaxations in uterine slices reflect uterine peristalsis *in vivo*. Significantly, the peristalsis frequency of uterine slices (see below) is close to that of the whole uterus *in vivo* (Crane and Martin, 1991; Meirzon et al., 2011; Zhang et al., 2019). Hence, we use the term “uterine peristalsis” to describe rhythmic contractions and relaxations in uterine slices from now on in this study.

To gain insight into the cellular signals responsible for uterine peristalsis in uterine slices, we measured Ca^2+^ signals with Cal-520 as Ca^2+^ is the primary signal for smooth muscle contraction. As shown in Figure 1B(i) and Supplementary Video 1, Ca^2+^ signals in the slices were not constant but oscillated. Moreover, Ca^2+^ oscillations were temporally associated with uterine peristalsis in a one-to-one manner [Figures 1B(i,ii),C and Supplementary Video 1]. Amplitudes of Ca^2+^ oscillations and changes in the uterine slice size showed a biphasic relationship (i.e., the two different fitting lines in two segments shown in Figures 1D,E). At the lower Ca^2+^ levels, there was a positive correlation indicated by the red fitting line between Ca^2+^ increase and the decrease in uterine area (*r* = 0.9659, *p* < 0.0001) when using the single event shown in Figure 1C (Figure 1D) and also when using all the events shown in Figure 1B (red fitting line, *r* = 0.8334, *p* < 0.0001) from the same slice (Figure 1E). But, at higher Ca^2+^ levels (i.e., those greater than 5% Δ*F*/*F*_0_ in this set of data), there were no correlations (two blue fitting lines; *r* = 0.2051, *p* = 0.1191, Figure 1D; *r* = 0.1117, *p* < 0.0075, Figure 1E). Visual inspection showed that this occurred when both Ca^2+^ oscillation frequency and contraction magnitudes reached and stayed at a plateau. The results in Figure 1 indicate that Ca^2+^ oscillations underlie uterine peristalsis.

To determine whether Ca^2+^ oscillations occurring only in USMCs cause uterine peristalsis, we studied uterine slices from acta2-R-CaMP1.07 mice. In these mice, acta2-R-CaMP1.07, a Ca^2+^ reporter, is expressed only in smooth muscle cells (Ohkura et al., 2012; Lu et al., 2021a). Supplementary Figure 2 and Supplementary Video 2 show that Ca^2+^ oscillations revealed by R-CaMP1.07 signals were temporally associated with uterine peristalsis, arguing that Ca^2+^ signals indeed originate from USMCs and cause the peristalsis.

### Pathological Features and Gene Expression Profile in Tamoxifen-Induced Adenomyotic Mice

**FIGURE 2:**
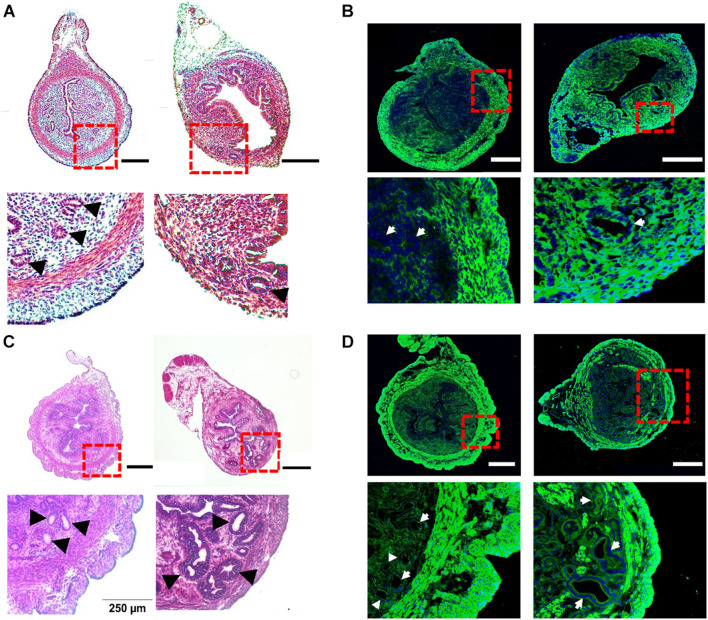
Histological characteristics of tamoxifen-induced adenomyosis in neonatal and adult mice. **(A)** The hematoxylin–eosin (H–E) staining of uterine cross-sections from postnatal day (PND) 14 mice after treatment with the vehicle for preparing tamoxifen (left) or with tamoxifen (right). Insets in **(A)** and other panels in this figure are enlarged areas in dotted red line boxes. **(B)** The immunostaining of Myh11 (green) in uteri from PND14 mice after treatment with the vehicle (left) or with tamoxifen (right). The nucleus, shown in blue in this panel (and in panel **D**), was stained with DAPI. **(C)** The H–E staining of uterine cross-sections from PND55 mice after treatment with the vehicle (left) or with tamoxifen (right). **(D)** The immunostaining of Myh11 (green) in uteri from PND55 mice after treatment with the vehicle (left) or with tamoxifen (right). Note that (1) glands as marked by black arrowheads **(A,C)** or white arrows **(B,D)** were present in the circular smooth muscle in both PND14 and PND55 mice after tamoxifen treatment, but they were absent in vehicle control mice of both ages, and (2) circular muscle in both PND14 and PND55 mice were intact without interruption in vehicle control mice but were disrupted by endometrial glands in mouse uteri treated with tamoxifen. *N* = 4–6 mice. Scale bar: 200 μm **(A,B)**; 500 μm **(C,D)**.

To investigate the potential changes in uterine peristalsis and Ca^2+^ signals in adenomyosis, we adapted a tamoxifen protocol developed by Parrott et al. (2001) to induce adenomyosis in CD-1 mice (Green et al., 2005; Mehasseb et al., 2009, 2010a). In control mice at PND14, the circular muscle layer was well developed to form an intact ring structure and did not contain endometrial tissues (Figure 2A, left). After treatment with tamoxifen, the circular muscle layer degenerated, with some muscle cells replaced with endometrial tissue and glands. However, the outer longitudinal layer appeared intact without invasion of endometrial tissue (Figure 2A, right). To examine the muscle layers’ changes, we immunostained uterine tissues with the antibody for Myh11, a smooth muscle-specific marker. As shown in Figure 2B (left), Myh11 positive cells formed a complete and intact circular ring and bundles in the longitudinal layer in control mice. However, in tamoxifen-treated mice, Myh11 positive cells in the circular muscle layer were interrupted by endometrial glands and stromal tissues, while we detected no such interruption in the longitudinal layer (Figure 2B, right).

In control mice at PND55, glands and muscle layers were more developed (Figure 2C) than PND14. Both glands and the circular muscle layer had clear boundaries, and no glands were detected in the circular muscle layer (Figure 2C, left). In tamoxifen-treated mice, the circular muscle layer degenerated further and was occupied and disrupted by variable sizes of endometrial glands (Figure 2C, right). These features were also evident when Myh11 was stained in both uterine slices (Figure 2D).

**FIGURE 3:**
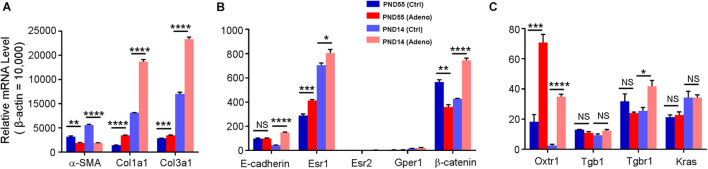
Changes in gene expression in adenomyotic uteri in neonatal and adult mice. mRNA for each gene was quantified by qPCR; the expression level was calculated using the 2^– Δ^ ^*Ct*^ method and was normalized against β–actin. Genes are grouped and plotted in panels **(A–C)**, according to their relative expression levels. Note that α-SMA, Col1a1, Col3a1, Esr1, and Oxtr1 were changed in the same direction in both age groups, while E-cadherin and Tgbr1 were up-regulated only at PND14, and β-catenin was changed in the opposite direction between two groups. N.S., no statistical significance, *P < 0.05, **P < 0.01, ***P < 0.001, ****P < 0.0001 with unpaired Student’s *t*-test (*N* = 3 repeats in **A–C** with 3 (adult) or 9 (neonate) mice in each repeat). Ctrl, vehicle treatment; Adeno, tamoxifen treatment; PND, postnatal day.

To further characterize the tamoxifen-induced adenomyosis model, we assessed the expression of genes that are potentially essential for uterine smooth muscle contraction (Wray, 1993; Bulletti et al., 2004; Sanborn, 2007; Young, 2007; Aguilar and Mitchell, 2010; Guerra and Hurt, 2019). The transcript for α-SMA, a primary structural protein in smooth muscle, was reduced by 41.38 ± 3.35% and 66.56 ± 0.88% at PND55 and PND14, respectively, in the tamoxifen-treated group compared to the control (Figure 3A). Among estrogen receptors, Esr1 expression was increased at both ages after treatment with tamoxifen, while Esr2 and Gper1 stayed low at both ages (Figure 3B). Oxytocin receptor was increased by 3.34 ± 0.89 fold at PND55 and 14.75 ± 4.88 fold at PND14 in the tamoxifen-treated group compared to the control group (Figure 3C).

We also measured the expression of the genes that may play a role in the pathogenesis of adenomyosis (Oh et al., 2013; Artymuk et al., 2019; Inoue et al., 2019; Kim et al., 2020; Adamyan et al., 2021). Kras and Tgb1 stayed at the same level in control and tamoxifen-treated mice at both ages (Figure 3C). Tgbr1 and β-catenin were reduced at PND55 while increased at PND14 in tamoxifen-treated mice compared to control mice (Figure 3C). E-cadherin expression was not different at PND55, but its level was increased by 2.39 ± 0.14 fold at PND14 (Figure 3B). Collagens 1 and 3 were increased in the tamoxifen-treated group at both ages compared to the control group (Figure 3A).

### Impairments in Embryo Implantation and Reproduction in Tamoxifen-Induced Adenomyotic Mice

**FIGURE 4:**
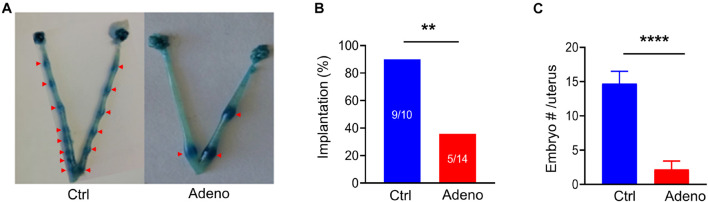
Adenomyotic mice exhibit abnormal embryo implantations. **(A)** Embryo implantations in uteri from a mouse after treatment with the vehicle for tamoxifen (Ctrl) (left) or a mouse after treatment with tamoxifen (Adeno) (right), as revealed by Chicago Blue dye staining. **(B)** Incidence of successful implantations in mice after treatment with the vehicle (Ctrl) or after tamoxifen treatment (Adeno). Implantation (%) = mice with successful embryos/total mating mice*100; **P < 0.01 by a Chi-Square test, *N* = 10 mice in the Ctrl group and 14 mice in the Adeno group. **(C)** Number of embryos in the Ctrl group and in the Adeno group. ****P < 0.0001 with unpaired Student’s *t*-test (*N* = 9 mice, Ctrl vs. 5 mice, Adeno).

To evaluate the impact of adenomyosis on reproduction, we measured the success rate of embryo implantation and the number of implanted embryos at 5 dpc (a vaginal plug as day 1) (Figure 4A). We found that the rate of embryo implantation was markedly decreased in tamoxifen-treated mice (Figure 4B). For those mice which had successful implantations, the number of implanted embryos was significantly smaller in tamoxifen-treated mice than in control mice (Figure 4C). Hence tamoxifen-induced adenomyosis causes mice to become less fertile.

### Spontaneous Uterine Peristalsis in Adult Adenomyotic Mice

**FIGURE 5:**
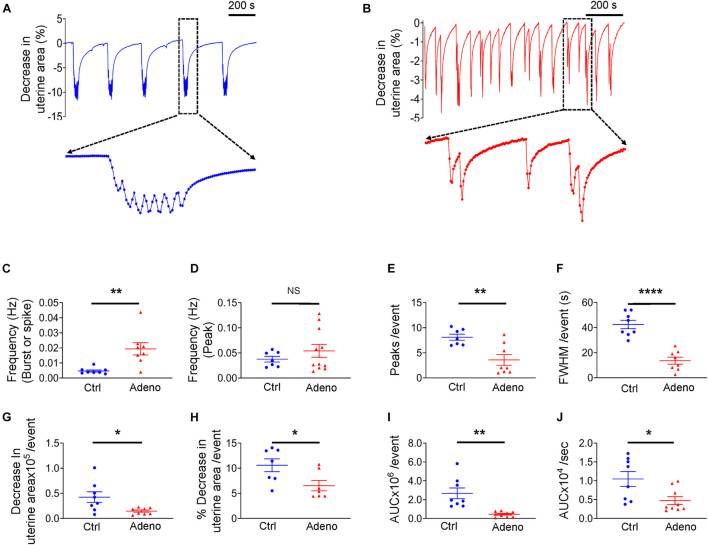
Changes in uterine peristalsis in adult adenomyotic mice. **(A)** Typical uterine peristalsis events in postnatal day (PND) 55 mice after treatment with the vehicle for tamoxifen (Ctrl). A burst event, marked with the dotted line box, is shown in an expanded time scale. **(B)** Typical uterine peristalsis events in PND55 mice after with tamoxifen (Adeno). Three events in the dotted line box are shown in an expanded time scale. **(C)** Frequency comparison between Ctrl mice and Adeno mice at PND55 when a burst or a spike was counted as one event (the same convention applied to panels **E–J**). **(D)** Frequency comparison between Ctrl mice and Adeno mice at PND55 when every peak was counted as one event. **(E)** Comparison of peak numbers/event between two group mice. **(F)** Full width at half maximum (FWHM)/event in two group mice. **(G)** Changes in uterine slice absolute areas/event in two group mice. **(H)** Changes in slice relative areas/event in two group mice. **(I)** Area under the curve (AUC)/event in two group mice. **(J)** Contraction intensity (AUC × Frequency) comparison between Ctrl and Adeno mice. Note that each data point in panels **(C–J)** is an average value from multiple slices in a mouse, and each slice was recorded for ∼1 h. N.S., no statistical significance, *P < 0.05, **P < 0.01, ****P < 0.0001 with unpaired Student’s *t*-test (*N* = 8 Ctrl mice (20–24 slices) and 8–11 Adeno mice (24–28 slices) for panels **(C–J).**

Having characterized the pathohistology of the mouse model of adenomyosis induced by tamoxifen, we next studied uterine peristalsis and Ca^2+^ signals in these mice using precision-cut uterine slices. Our initial preliminary experiments noted that the Ca^2+^ indicator, Cal-520, mildly altered the uterine peristalsis pattern. Therefore, to better determine uterine peristalsis characteristics in normal and adenomyotic mice, we examined uterine peristalsis without Cal-520. Figures 5A,B show typical recordings of uterine peristalsis in control CD-1 mice and adenomyotic CD-1 mice at PND55. Of note, in control mice, uterine peristalsis occurred as bursts that consisted of multiple peaks (Figure 5A). However, in adenomyotic mice, uterine peristalsis appeared as spikes with a single peak or bursts with a few peaks (Figure 5B). Given the unique contractile patterns of uterine peristalsis, we analyzed the uterine peristalsis frequency in two ways. One is to count a burst or a spike as one peristalsis event, and the other an individual peak within bursts or in spikes as one event. When counting based on the burst and spike, uterine peristalsis frequency was significantly increased in adenomyotic mice compared with control mice (Figure 5C). However, when counting based on the peak, uterine peristalsis frequency was not significantly different between the two groups (Figure 5D). On average, peristaltic burst in control mice oscillated 7.57 ± 0.51 times, while in adenomyotic mice, this value was 4.13 ± 0.73 with over 50% of events presenting as spikes (i.e., with just one peak) (Figure 5E). As expected, full width at half maximum (FWHM) of the uterine peristalsis was significantly longer in control mice than in adenomyotic mice when bursts or spikes were counted as events (Figure 5F).

Force generated by uterine peristalsis should be proportional to the change in the uterine area. We found that change in uterine area per uterine peristalsis (with the burst or spike as the event) was smaller in adenomyotic uterine slices than in control slices in terms of an absolute value (Figure 5G) or a relative value (Figure 5H). To further quantify the change in force, we calculated the product of frequency (Figure 5C) and area under the curve (AUC), the area between the curve and the *x*-axis (Figure 5I), designated as contraction intensity (Figure 5J). Figure 5J reveals that contraction intensity was weaker in adenomyotic uterine slices than in control slices.

The above analyses indicate that uterine peristalsis activates as bursts in normal adult mice, while it turns into spikes in adenomyotic mature mice. Overall, uterine peristalsis in adult adenomyotic mice produces weaker contractions than in normal adult mice.

### Spontaneous Ca^2+^ Signals in Adult Adenomyotic Mice

**FIGURE 6:**
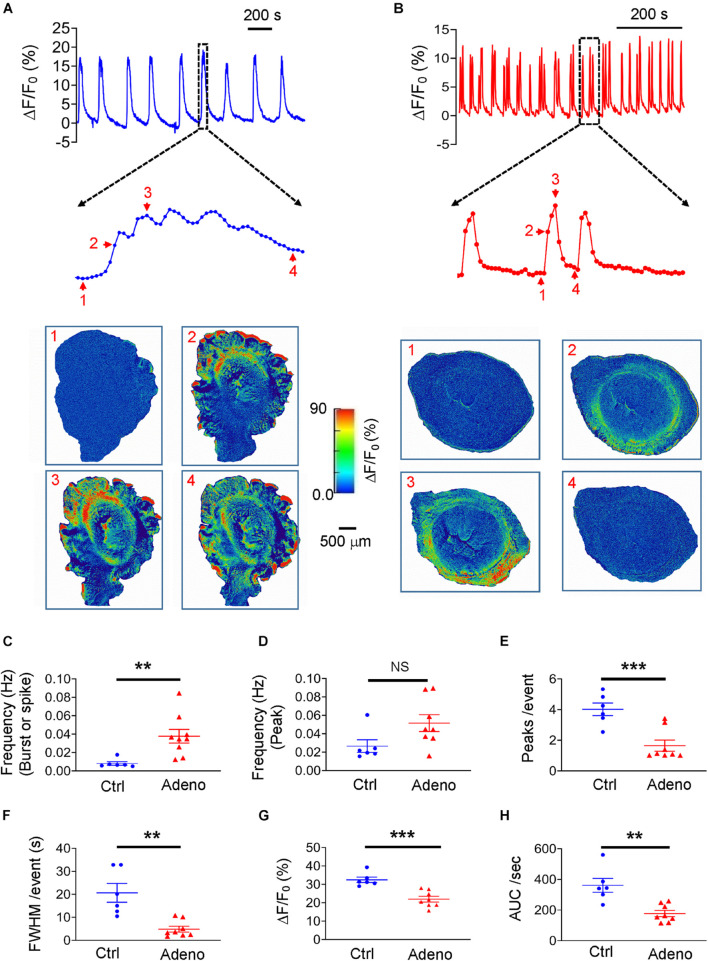
Changes in Ca^2+^ oscillations in adult adenomyotic mice. **(A)** Typical Ca^2+^ oscillations in postnatal day (PND) 55 mice after treatment with the vehicle for tamoxifen (Ctrl). A burst event as marked with the dotted line box is shown in an expanded time scale, and images below the trace are snapshots of this event as indicated by the corresponding numbers near the trace. **(B)** Typical Ca^2+^ oscillations in PND55 mice after treatment with tamoxifen. Three events as marked with the dotted line box are shown in an expanded time scale, and images below the trace are snapshots of one of these events, as shown by the corresponding numbers near the trace. **(C)** Ca^2+^ oscillation frequency when a burst or a spike was counted as one event (the same convention is applied to panels **E–H**). **(D)** Ca^2+^ oscillation frequency when an individual peak in bursts or spikes was counted as an event. **(E)** Numbers of Ca^2+^ oscillations per event in uterine slices from Ctrl and Adeno mice at PND55. **(F)** Full width at half maximum (FWHM)/event in uterine slices from Ctrl and Adeno mice at PND55. **(G)** Amplitude of Ca^2+^ oscillations in uterine slices from Ctrl and Adeno mice at PND55. **(H)** Calcium intensity (AUC × Frequency) in uterine slices from Ctrl and Adeno mice at PND55. Note that each data point in panels **(C–H)** is an average value from multiple slices in a mouse, and each slice was recorded for 30 min. N.S., no statistical significance, **P < 0.01, ***P < 0.001 with unpaired Student’s *t*-test [*N* = 6 Ctrl mice (15 slices) and 9 Adeno mice (23–26 slices) for panels **C–H**].

We loaded uterine slices with Cal-520 to get insight into the characteristics of Ca^2+^ oscillations underlying normal and dysfunctional uterine peristalsis. As shown in Figures 6A,B where typical Ca^2+^ oscillations from mature control mice and adenomyotic mice are displayed, Ca^2+^ oscillations exhibited as bursts in control slices but as spikes in adenomyotic slices. When counting a burst or a spike as an event, the frequency of Ca^2+^ oscillations was higher in adenomyotic slices than in control slices (Figure 6C). However, when counting based on the peaks, the frequency of Ca^2+^ oscillations was not statistically different in both control slices and adenomyotic slices (Figure 6D). The number of oscillations per event (Figure 6E) and FWHM (Figure 6F) in the slices from adult adenomyotic mice were smaller than those from control mice.

We further determined the amplitude of Ca^2+^ oscillations in both types of uterine slices. We found that amplitude with Δ*F*/*F*_0_ as a proxy of intracellular Ca^2+^ concentration ([Ca^2+^]_*i*_) was smaller in adenomyotic slices than in control slices (Figure 6G). Finally, we calculated the product of frequency and AUC, designated as calcium intensity. As shown in Figure 6H, the calcium intensity was significantly smaller in adenomyotic slices than control slices. These results indicate that Ca^2+^ oscillations change from bursts in adult control uteri into spikes with weaker intensity in adult adenomyotic uteri, consistent with uterine peristalsis features in Figure 5.

### Spontaneous Uterine Peristalsis in Neonatal Adenomyotic Mice

**FIGURE 7:**
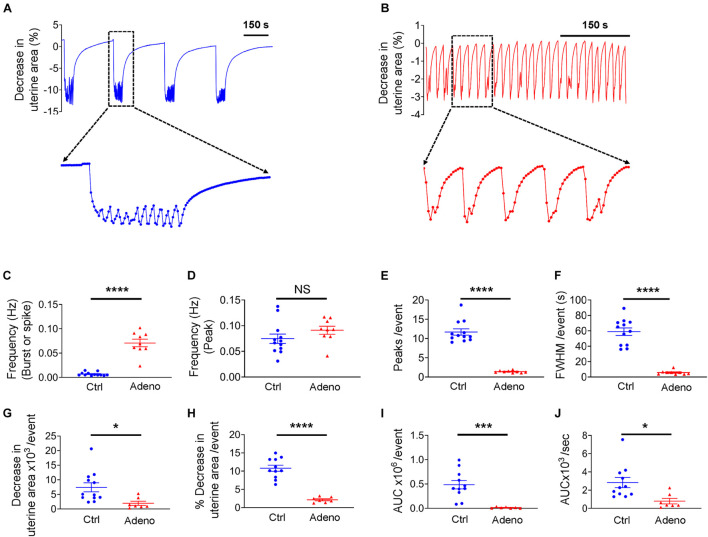
Changes in uterine peristalsis in neonatal adenomyotic mice. **(A)** Typical peristalsis events in uterine slices from postnatal day (PND) 14 mice after treatment with the vehicle for tamoxifen (Ctrl). A burst event as marked with the dotted line box is shown in an expanded time scale. **(B)** Typical peristalsis events in uterine slices from PND14 mice after treatment with tamoxifen (Adeno). Five events marked with the dotted line box are shown in an expanded time scale. **(C)** Frequency comparison between Ctrl mice and Adeno mice at PND14 when a burst or a spike was counted as an event (the same convention is applied to panels **E–J**). **(D)** Frequency comparison between Ctrl mice and adeno mice at PND14 when a peak was counted as an event. **(E)** Comparison on peak numbers/event between Ctrl and Adeno mice at PND14. **(F)** Full width at half maximum (FWHM)/event in Ctrl and Adeno mice at PND14. **(G)** Changes in uterine slice absolute areas/event in Ctrl and Adeno mice at PND14. **(H)** Changes in slice relative areas/event in control and adenomyotic mice. **(I)** AUC/event in Ctrl and Adeno mice at PND14. **(J)** Contraction intensity (AUC × Frequency) comparison between Ctrl and Adeno mice at PND14. Note that each data point in panels **(C–J)** is an average value from multiple slices in a mouse, and each slice was recorded for ∼1 h. N.S., no statistical significance, *P < 0.05, ***P < 0.001, ****P < 0.0001 with unpaired Student’s *t*-test [*N* = 11 Ctrl mice (40–44 slices) and 9 Adeno mice (24–29 slices) for panels **C–J**].

Uterine histoarchitecture is well established at PND 14 in mice (Figure 2A). However, to the best of our knowledge, no study on uterine peristalsis at this stage in normal mice and adenomyotic mice exists. Figure 7A shows typical uterine peristalsis at low and high temporal resolutions from a neonatal control mouse. Uterine peristalsis rose quickly and was maintained at a plateau with small oscillations. Visual inspection of the uterine slice indicates that the sustained plateau resulted from sequential contractions. The frequency of uterine peristalsis at this stage was 0.0067 ± 0.00062 Hz when each burst or spike was counted as one event (Figure 7C). This value was 0.075 ± 0.0051 Hz when each peak was counted as one event (Figure 7D). On average, a burst was the sum of 12.16 ± 0.55 individual contractions (Figure 7E), lasting for 60.57 ± 3.34 seconds at FWHM (Figure 7F).

In adenomyotic mice at PND 14, uterine peristalsis comprised a single contraction or occasional multiple contractions, as shown in both low and high temporal resolution recordings (Figure 7B). Overall, uterine peristalsis at PND14 in adenomyotic mice comprised many fewer contractions (Figure 7E) and lasted a much shorter time, as assessed by FWHM (Figure 7F), when compared to control mice. Uterine peristalsis frequency was higher in adenomyotic mice than in control mice (Figure 7C) when a burst or a spike was counted as an event. However, there was no difference when the frequency calculation was based peak numbers (Figure 7D).

To assess the force generated by uterine peristalsis, we compared changes in the uterine area and contraction intensity between control mice and adenomyotic mice at PND14. Both uterine area changes in either absolute terms (Figure 7G) or relative terms (Figure 7H) were smaller in adenomyotic mice than in control mice. The AUC (Figure 7I) and contraction intensity (Figure 7J) caused by uterine peristalsis were smaller and weaker, respectively, in adenomyotic mice than in control mice.

### Spontaneous Ca^2+^ Signals in Neonatal Adenomyotic Mice

**FIGURE 8:**
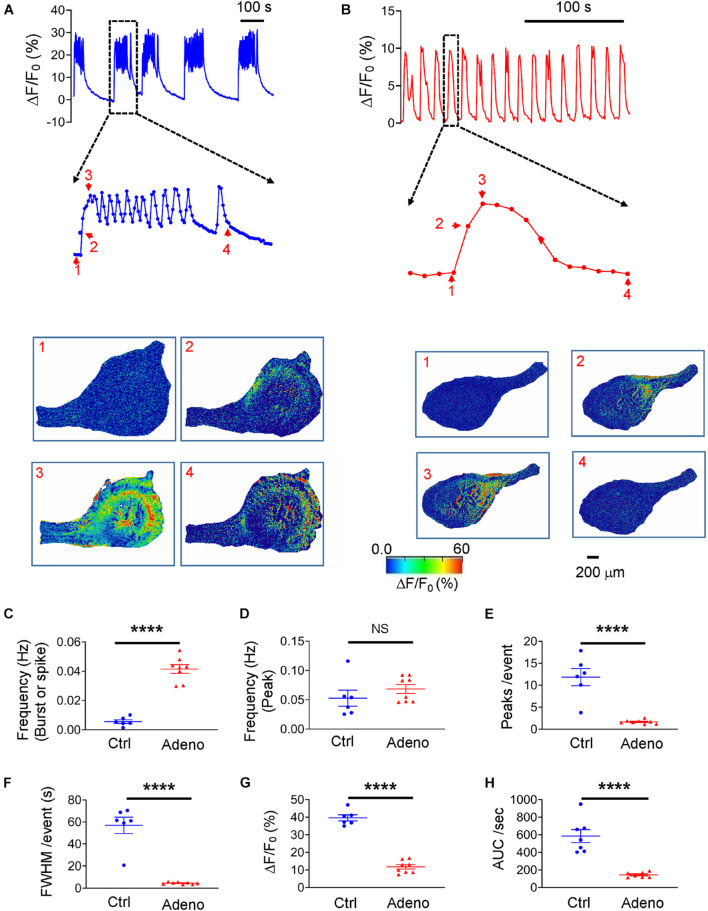
Changes in spontaneous Ca^2+^ oscillations in neonatal adenomyotic mice. **(A)** Typical Ca^2+^ oscillations in uterine slices from postnatal day (PND) 14 mice after treatment with the vehicle for tamoxifen (Ctrl). A burst event marked with the dotted line box is shown in an expanded time scale, and images below the trace are snapshots of this event as indicated by the corresponding numbers near the trace. **(B)** Typical Ca^2+^ oscillations in uterine slices from PND14 mice after treatment with tamoxifen. An event marked with the dotted line box is shown in an expanded time scale, and images below the trace are snapshots of this event, as shown by the corresponding numbers near the trace. **(C)** Ca^2+^ oscillation frequency when a burst or a spike was counted as an event (the same convention is applied to panels **E–H**). **(D)** Ca^2+^ oscillation frequency when a peak in bursts or spikes was counted as an event. **(E)** Numbers of Ca^2+^ oscillations per event in Ctrl and Adeno mice at PND14. **(F)** Full width at half maximum (FWHM)/event in Ctrl and Adeno mice at PND14. **(G)** Amplitude of Ca^2+^ oscillations in Ctrl and Adeno mice at PND14. **(H)** Calcium intensity (AUC × Frequency) in Ctrl and Adeno mice at PND14. Note that each data point in panels **C–H** is an average value from multiple slices in a mouse, and each slice was recorded for 30 min. N.S., no statistical significance, ****P < 0.0001 with unpaired Student’s *t*-test [*N* = 6 Ctrl mice (14–17 slices) and 8 Adeno mice (18–23 slices) for panels **C–H**].

Loading the slices with Cal-520 enabled us to measure Ca^2+^ signals corresponding to uterine peristalsis in control and adenomyotic mice at PND14. Ca^2+^ signals exhibited as bursts in control mice (Figure 8A). In adenomyotic mice, Ca^2+^ signals were largely spikes with a quick rise and a slower decay (Figure 8B). When a burst or a spike was counted as an event, Ca^2+^ oscillations had a higher frequency (Figure 8C) and shorter FWHM (Figure 8F) in adenomyotic mice than in control mice. When counting events based on the peak, Ca^2+^ oscillation frequency was not significantly different between control and adenomyotic groups (Figure 8D); Each burst comprised 11.97 ± 1.43 oscillations in control mice and 1.66 ± 0.15 in adenomyotic mice (Figure 8E). Δ*F*/*F*_0_, i.e., [Ca^2+^]_*i*_, and calcium intensity were smaller in adenomyotic mice than in control mice at PND14 (Figures 8G,H).

### Oxytocin-Induced Changes in Ca^2+^ Signals and Uterine Peristalsis in Adult Adenomyotic Mice

**FIGURE 9:**
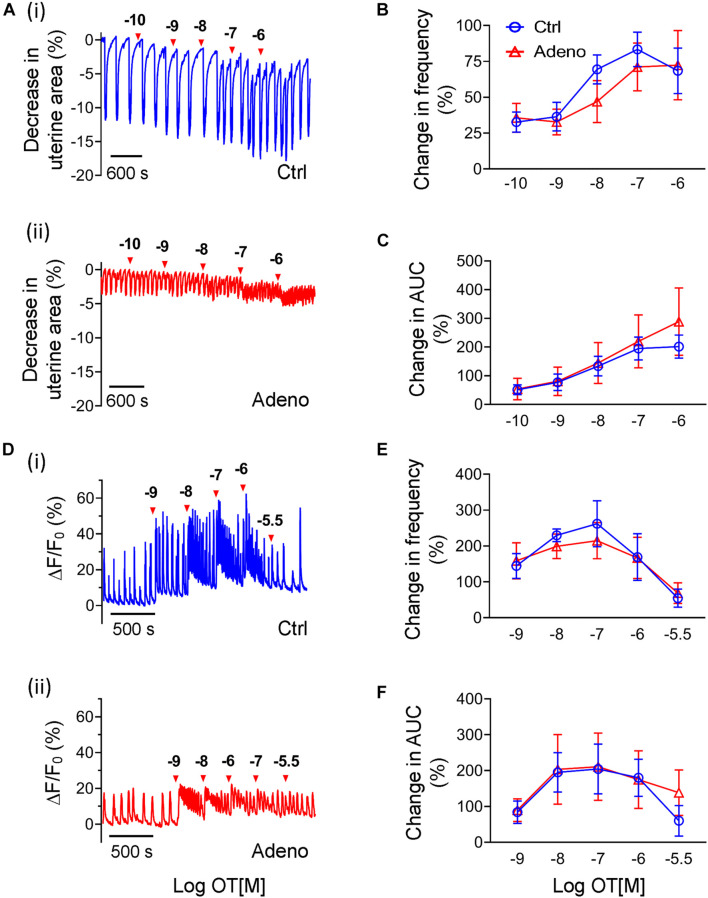
Changes in oxytocin-induced uterine peristalsis and Ca^2+^ oscillations in adult adenomyotic mice. **(A)** Time courses of uterine peristalsis in response to accumulative oxytocin (OT) stimulation in postnatal day (PND) 55 mice after treatment with the vehicle for tamoxifen (Ctrl) **[A(i)]** and the same age mice after treatment with tamoxifen (Adeno) **[A(ii)]**. The concentrations of oxytocin are labeled above the traces. **(B)** Uterine peristalsis frequency changes caused by oxytocin in uterine slices from Ctrl (O; blue line) and Adeno (Δ; red line) mice at PND55. **(C)** Changes in the AUC of uterine peristalsis caused by OT in uterine slices from Ctrl (O) and Adeno (Δ) mice at PND55. **(D)** Time courses of Ca^2+^ oscillations in response to accumulative OT stimulation in Ctrl **[D(i)]** and Adeno mice **[D(ii)]** at PND55. The concentrations of OT are labeled above the traces. **(E)** Ca^2+^ oscillation frequency changes caused by OT in uterine slices from Ctrl (O; blue line) and adeno (Δ; red line) mice at PND55. **(F)** Change in the AUC of Ca^2+^ oscillations by OT in uterine slices from Ctrl (O; blue line) and Adeno (Δ; red line) mice at PND55. Note that a burst or a spike was counted as an event in this Figure (and Figure 10). *N* = 5 Ctrl mice (10 slices) and 5 Adeno mice (10 slices). A two-way ANOVA to compare individual groups shows no significant difference in panels **(B,C,E,F)**.

Oxytocin contributes to adenomyosis as proposed by the ‘tissue injury and repair’ hypothesis (Nie et al., 2010; Leyendecker and Wildt, 2011), hence we examined whether oxytocin affects Ca^2+^ signals and uterine peristalsis. At PND55, oxytocin increased the frequency of uterine peristalsis and Ca^2+^ oscillations in a dose-dependent manner in control mice and adenomyosis mice when bursts or spikes were used for event counting (Figure 9). For uterine peristalsis, oxytocin increased its frequency throughout the dose range tested (Figure 9B), but for Ca^2+^ responses, oxytocin at 0.1 μM yielded the maximal increase (Figure 9E). However, there were no significant differences in both responses between two groups (Figures 9B,E).

Besides frequency, oxytocin also increased amplitudes of uterine peristalsis and Ca^2+^ oscillations. Because oxytocin changed the signals’ baseline, we calculated AUCs to reflect changes in amplitude of both responses by oxytocin. We found that oxytocin dose-dependently increased the AUCs of uterine peristalsis (Figure 9C) and Ca^2+^ signals (Figure 9F). However, we found no difference in both responses between control mice and adenomyotic mice at PND55 (Figures 9C,F).

### Oxytocin-Induced Changes in Ca^2+^ Signals and Peristalsis in Neonatal Adenomyotic Mice

**FIGURE 10:**
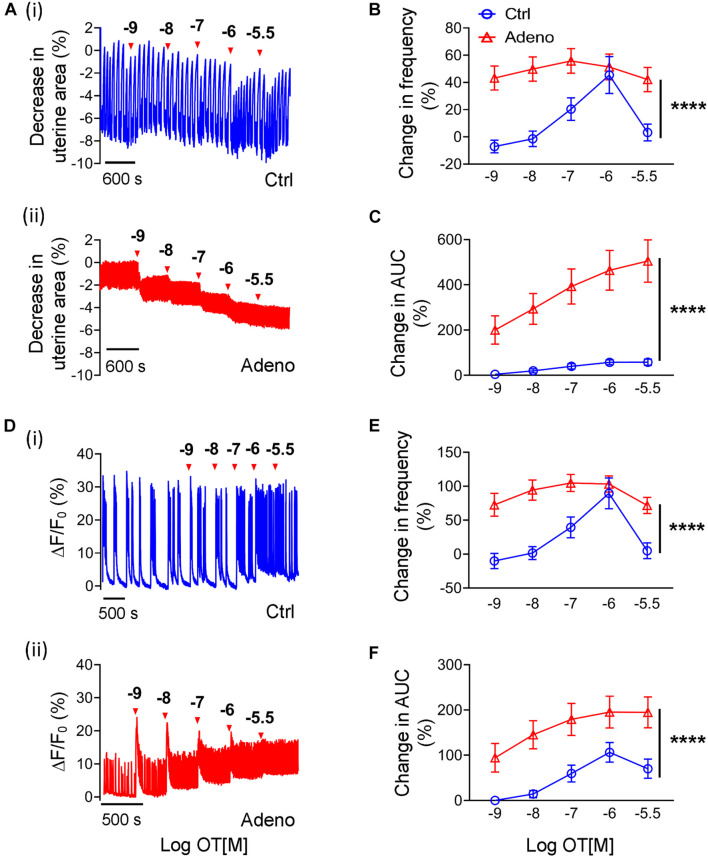
Changes in oxytocin-induced uterine peristalsis and Ca^2+^ oscillations in neonatal adenomyotic mice. **(A)** Time courses of uterine peristalsis in response to accumulative oxytocin (OT) stimulation in postnatal day (PND) 14 mice after treatment with the vehicle for tamoxifen (Ctrl) **[A(i)]** and the same age mice after treatment with tamoxifen (Adeno) **[A(ii)]**. The concentrations of OT are labeled above the traces. **(B)** Uterine peristalsis frequency changes caused by OT in uterine slices from Ctrl (O; blue line) and Adeno (Δ; red line) mice at PND14. **(C)** Changes in the AUC of uterine peristalsis by OT in uterine slices from Ctrl (O; blue line) and Adeno (Δ; red line) mice at PND14. **(D)** Time courses of Ca^2+^ oscillations in response to accumulative OT stimulation in Ctrl **[D(i)]** and Adeno mice **[D(ii)]** at PND14. The concentrations of OT are labeled above the traces. **(E)** Ca^2+^ oscillation frequency changes caused by OT in uterine slices from Ctrl (O; blue line) and Adeno (Δ; red line) mice at PND14. **(F)** OT-induced changes in the AUC of Ca^2+^ oscillations in uterine slices from Ctrl (O; blue line) and Adeno (Δ; red line) mice at PND14. *N* = 5 Ctrl mice (10 slices) and 5 Adeno mice (10 slices) from for panels **(B,C,E,F)**. ****P < 0.0001 by a two-way ANOVA.

As there is no study on oxytocin’s effect on uterus’s contractile responses in neonatal mice, we studied them. Figure 10A shows original traces of uterine peristalsis in response to oxytocin in control mice and adenomyosis mice at PND14. In contrast to adult mice at PND55, there was a striking difference in response to oxytocin between control mice and adenomyotic mice at PND14. In control mice, oxytocin at concentrations between 10^–9^ to 3 × 10^–5^ M only modestly increased uterine peristalsis frequency and AUC (Figures 10B,C). However, in adenomyotic mice, oxytocin at 10^–9^ M caused a marked increase in uterine peristalsis both in terms of frequency and AUC. This effect was dose-dependent within the range of 10^–9^ to 3 × 10^–6^ M (Figures 10B,C).

We further examined Ca^2+^ signals in response to oxytocin in control and adenomyotic mice at PND14 (Figure 10D). Like uterine peristalsis, oxytocin increased the frequency of Ca^2+^ oscillations in a dose-dependent manner in both control and adenomyosis mice (Figure 10E). However, it caused these oscillations at lower concentrations in adenomyotic mice. It also increased the oscillations more in adenomyotic mice than in control mice (Figures 10E,F). Both Ca^2+^ signals and uterine peristalsis analyses show that adenomyotic uteri at PND14 became hypersensitive to oxytocin.

## Discussion

Uterine peristalsis is a biomechanical activity critical for reproduction and fertility, and its dysfunction has pervasive implications in adenomyosis. Hence, a better understanding of this activity in healthy and adenomyotic conditions is important in advancing reproductive biology and finding therapeutic strategies to manage this gynecological disorder. This study developed a uterine slice preparation with several unique features, permitting uterine peristalsis to be quantified in unprecedented details. First, uterine slices preserve the same complex cell architecture and maintain the local cell–cell and cell–matrix contacts seen *in vivo*. This is significant and one prerequisite for an objective study of uterine peristalsis and its role in adenomyosis because peristalsis is a tissue activity involving multiple cells acting in synchronization, and adenomyosis involves both endometrium and myometrium. Second, as revealed by *in vivo* studies (van Gestel et al., 2003; Kissler et al., 2004; Lesny and Killick, 2004; Fanchin and Ayoubi, 2009), uterine peristalsis results from the contraction and relaxation of the circular smooth muscle. Therefore, uterine slices prepared using cross-sections (as in this study) permit peristalsis like that seen *in vivo*. And third, uterine slices’ thickness is well suited for imaging, allowing one to gain spatial information about the peristalsis and its underlying signals. In this study, we found that Ca^2+^ oscillations and uterine peristalsis occur in a one-to-one fashion, and their amplitudes are positively correlated, consistent with the hypothesis that oscillating Ca^2+^ changes cause uterine peristalsis. In the future, two-photon microscopy will allow Ca^2+^ signals and resulting peristalsis to be viewed at cellular and subcellular resolutions [as we did with anal sphincter slices (Lu et al., 2021a)]. However, we do caution not to over- extrapolate the phenomena observed in the slices to the entire uterus *in vivo* for at least two reasons. First the connections between circular muscle bundles that may be critical for uterine peristalsis propagation *in vivo* may be lost or disrupted in the slices. And secondly, the force from the longitudinal muscle contraction in the slices does not decrease the uterine slice size, but *in vivo* this force can change uterine contraction and peristaltic waves at least during the menstruation in humans (Martinez-Gaudio et al., 1973; de Vries et al., 1990).

With the uterine slice preparation, we showed that uterine peristalsis in normal mice exhibits bursts because of Ca^2+^ oscillations in circular smooth muscle cells. In contrast, in adenomyotic mice, uterine peristalsis and Ca^2+^ signals display predominantly as single peak spikes. The frequency of uterine peristalsis or Ca^2+^ oscillations, when calculated based on the number of peaks, is not significantly different between adenomyotic mice and control mice (in adult or neonatal mice, Figures 5–8). However, the estimated contraction and Ca^2+^ intensities are lower in adenomyotic mice than in control mice. These results fill an important gap in our understanding of uterine peristalsis in adenomyosis, as how uterine peristalsis is changed in this disorder remain unclear. MRI and ultrasound studies suggest that uterine peristalsis frequency in adenomyosis patients is higher than in healthy women (Leyendecker et al., 1996; Leyendecker and Wildt, 2011). However, these methods could not provide information about the peristalsis’ amplitude and the force generated by peristalsis, neither do they have temporal resolution to differentiate burst and spike uterine contraction. An alternative method to determine these parameters has been to measure uterine contraction using isolated uterine strips under isometric configurations. With this technique, Zhu et al. (2015) reported that uteri from adenomyotic mice generate stronger spontaneous contractions than normal subjects. This functional result, however, is not supported by immunohistology and gene expression results. As shown in this study and others (Parrott et al., 2001; Mehasseb et al., 2009, 2010a), the circular muscle in adenomyotic uteri in mice becomes disorganized and degenerate with less contractile proteins such as Myh11 and α-SMA. Similarly, circular smooth muscle disarray is also a characteristic in human adenomyotic uteri (Mehasseb et al., 2010b, 2011; Ibrahim et al., 2015). These histological and gene profile data are more in line with our finding that Ca^2+^ signals and uterine peristalsis are weaker in adenomyotic uteri than in healthy uteri. One potential reason for the discrepancy in the uterine contraction between this study and Zhu et al.’s (2015) is that tamoxifen-treated mice at PND14 and 55 were studied here, while mice at 4 months after tamoxifen were used in Zhu et al.’s (2015). However, Green et al. (2005) found that uterine hypoplasia after tamoxifen dosing in CD-1 mice is persistent up to 9 months with disorganized fascicles of smooth muscle and increased interstitial collagen. Likely, uterine peristalsis in this 9-month stage of mice would also be disrupted–contracting with a weaker force. Whether our observations will be true in human adenomyosis warrants further investigation. It is interesting that large- conductance Ca^2+^-activated K^+^ (BK_*Ca*_) channels and voltage-gated K^+^ channels are up-regulated in myometrial cells from patients with adenomyosis (Shi et al., 2016). These changes could in turn suppress uterine smooth muscle contraction (Anwer et al., 1993; Smith et al., 2007; Li et al., 2014; Lorca et al., 2014; Villegas et al., 2021).

Another main finding in this study is that neonatal and adult mice show unique characteristics in Ca^2+^ oscillations and uterine peristalsis. Regardless of the event counting methods used (C and D in Figures 5–8), both Ca^2+^ oscillations and uterine peristalsis are more active in neonatal mice than in adult mice. Moreover, oxytocin increases uterine peristalsis and Ca^2+^ oscillations in neonatal adenomyotic mice but not in adult adenomyotic mice compared to their age-matched controls (Figures 9, 10). It is known that uterine organogenesis in mice is complete at birth (Spencer et al., 2012). However, its histoarchitecture is only established and completed postnatally. It is not until PND10 that uterine glands bud and extend from the luminal epithelium into the surrounding endometrial stroma. As Ca^2+^ signals and mechanical force are two major factors controlling tissue growth and development (Inman and Smutny, 2021; Levin, 2021), it is likely that more active Ca^2+^ signals and uterine peristalsis in the neonatal stage facilitate the transition of the uterus to the adult configuration. This, in turn, may make the uterus at this stage more prone to damage or disruption by intrinsic and extrinsic factors. Therefore, the fact that oxytocin increases Ca^2+^ oscillations and uterine peristalsis in the neonatal stage but not in the adult stage implies oxytocin may regulate these two signals to contribute to the adenomyosis initiation and progression, which is consistent with the role of oxytocin proposed in the tissue injury and repair hypothesis for adenomyosis. Moreover, our study reveals that the regulation of oxytocin receptor expression by tamoxifen treatment is at least one of the molecular mechanisms underlying the oxytocin-induced increase in Ca^2+^ oscillations and uterine peristalsis during the neonatal stage. However, it is unexpected that oxytocin’s effects on uterine peristalsis and Ca^2+^ oscillation are not different between control and adenomyotic adult mice because the oxytocin receptor is also up-regulated in adult mice (although to a lesser degree than at the neonatal stage). Potentially, this level of increase in oxytocin receptor expression is not enough to exert meaningful contraction changes in adult mice.

What might be the implications of our findings on uterine peristalsis on the etiology of adenomyosis? In a recent modeling study of uterine peristalsis and adenomyosis, Shaked et al. (2015) found that a decrease in contraction wavelength and increased wave frequency lead to high levels of stress near the inner uterine cavity (Shaked et al., 2015). Our finding that uterine peristalsis switches from low frequency bursts to high frequency spikes in adenomyotic mice provides direct experimental evidence to support the hypothesis that uterine peristalsis can be a causal factor for adenomyosis. Our finding also raises the possibility that the uterine peristalsis pattern might play a more critical role than the total force generated by uterine peristalsis in the pathogenesis of adenomyosis. Finally, hyper-responsiveness to oxytocin in the neonatal stage but not in the adult stage upon tamoxifen treatment implies that uterine peristalsis could be a causal signal which initiates adenomyosis. Alternatively, changes in uterine peristalsis may not be an initial causal factor for adenomyosis, instead they may act as a downstream step of those initial casual factors, essential for the progression of the adenomyosis. It is also likely that these changes in uterine peristalsis are merely the consequence of the adenomyosis. However, even if this is the case, these changes may underlie many symptoms of adenomyosis such as dysmenorrhea and chronic pelvic pain (Vannuccini et al., 2017; Chiang et al., 2020; Zhai et al., 2020).

Tamoxifen-induced adenomyosis in mice is a well-established animal model for this disease. However, essentially no studies have examined the change in reproduction in this mouse model. We found that these mice either didn’t have embryos implanted or they implanted with significantly fewer embryos. These results replicate the clinical observation that adenomyosis patients often are sub-fertile and suffer more miscarriages. They may offer direct guidance to human adenomyosis patients as post-menopausal women with breast cancer treated with tamoxifen have a higher adenomyosis rate than those untreated (Cohen et al., 1997), and endocrine disruptors like tamoxifen are one of the underlying causes for the rising in adenomyosis cases globally (Smarr et al., 2016; Ho et al., 2017). Given that uteri from tamoxifen-treated mice undergo a mode switch of peristalsis in mice, one may speculate that such a switch may interfere with the fertilized egg or embryo transplantation inside the reproductive tract, leading to lowered fertility and miscarriages in adenomyosis patients. We should point out that the neonatal exposure to tamoxifen causes a broad range of effects on uterine and other reproductive organs that can last through adulthood. These effects may interfere with ovulation, fertilization, sperm and embryo transportation, or the receptivity of endometrium in adult mice. A severe interruption in one of these processes could also contribute to the lower number of embryo implantation sites seen in adenomyotic mice. More investigation into these possibilities in this mouse model of adenomyosis are warranted.

This study points to several future lines of investigation on uterine peristalsis in the non-pregnant uterus, a research area that has been neglected, yet is of clinical importance (Ma et al., 2020; Ivell and Anand-Ivell, 2021). A straight-forward and obvious investigation would study the molecular basis underlying the mode switch of uterine Ca^2+^ oscillations and peristalsis. Another closely related study might examine the molecular mechanisms underpinning the generation of Ca^2+^ oscillations and uterine peristalsis. Advancements in these areas should improve our understanding of how uterine dysperistalsis facilitates endometrial glands’ invasion into the myometrium. We believe that uterine slices from mice offer an attractive avenue to these lines of inquiry, as genes in mice can be readily modified. The third line of inquiry would explore how uterine peristalsis affects endometriosis and uterine fibroids’ pathogenesis. Even with mild-to-moderate disease, women with endometriosis are thought to significantly increase peristaltic contractions through the entire follicular phase and mid-luteal phase (Leyendecker et al., 2004). MRI wave motion studies in small numbers of women with fibroids detect focal myometrial wave motion next to fibrotic tissue in women with submucosal fibroids but not in intramural or subserosal located fibroids (Nishino et al., 2005). Therefore, studying uterine peristalsis in uterine slices from animal models of endometriosis and uterine fibroids (or studying slices from patients with these disorders) should shed light on these gynecological diseases’ pathogenesis.

In conclusion, we developed a uterine preparation that keeps the cell arrangement as it exists *in vivo* and generates uterine peristalsis as seen in the whole uterus *in situ*. With Ca^2+^ imaging, we discovered that Ca^2+^ oscillations are the underlying signals which induce uterine peristalsis. We further revealed that Ca^2+^ oscillations and peristaltic contractions switch from compound “burst” events to spikes without changing overall peak frequency. Moreover, oxytocin increases Ca^2+^ signals and uterine peristalsis to a greater extent in adenomyotic uteri than in healthy uteri in neonates but not in adults. These two changes suggest that dysfunction in uterine Ca^2+^ oscillations and peristalsis may act as a causal factor of adenomyosis and impair embryo implantation, potentially leading to lowered fertility in adenomyosis. The preparation and methodologies developed in this study provide a unique opportunity to further explore the molecular basis of uterine peristalsis and its potential role in adenomyosis and other genealogical diseases where uterine peristalsis may play a role.

## Data Availability Statement

The original data presented in the study are included in the article/Supplementary Material; further inquiries can be directed to the corresponding author.

## Ethics Statement

The animal study was reviewed and approved by UMMS IACUC.

## Author Contributions

MQ and PL designed and performed the experiments. MQ, PL, KB, and LL analyzed the data and/or performed statistical analysis. RZ conceived the study, designed the experiments, analyzed the data, and wrote the manuscript with contributions by MQ, PL, and LL. All authors reviewed and approved of the final manuscript.

## Conflict of Interest

The authors declare that the research was conducted in the absence of any commercial or financial relationships that could be construed as a potential conflict of interest.

## Publisher’s Note

All claims expressed in this article are solely those of the authors and do not necessarily represent those of their affiliated organizations, or those of the publisher, the editors and the reviewers. Any product that may be evaluated in this article, or claim that may be made by its manufacturer, is not guaranteed or endorsed by the publisher.

## Footnotes

1
https://imagej.nih.gov/ij/

## Supplementary Material

Supplementary Figure 1 Uterine peristalsis causes a repetitive spatio-temporal change in uterine slices as revealed by reslicing through the two marked lines to produce kymographs.

Supplementary Figure 2 Ca^2+^ signals as revealed by the R-CaMP1.07 Ca^2+^ reporter showing a close temporal association between Ca^2+^ oscillations and uterine peristalsis. R-CaMP1.07 fluorescence was recorded with a 4× objective (Nikon, Tokyo, Japan) at a speed of 3 Hz. The 568 nm line of an argon-ion laser provided fluorescence excitation, with a shutter to control exposure duration; emission of the Ca^2+^ indicator was monitored at wavelengths >600 nm. Changes in uterine area and fluorescence were calculated as described under the methods and materials section.

Supplementary Video 1 The movie clip showing the original (raw fluorescence) data for in Figure 1C; Time: Sec.

Supplementary Video 2 The movie clip showing the original (raw fluorescence) data for the event marked by the red arrow in Supplementary Figure 2; Time: Sec.

Supplementary Video 3 The movie clip showing the original data for the events in the dotted box in Figure 5A; Time: Sec.

Supplementary Video 4 The movie clip showing the original data for the events in the dotted box in Figure 5B; Time: Sec.

Supplementary Video 5 The movie clip showing the original data for the events in the dotted box in Figure 7A; Time: Sec.

Supplementary Video 6 The movie clip showing the original data for the events in the dotted box in Figure 7B; Time: Sec.
