# Supplementary figures and images for: Mode Switch of Ca2 + Oscillation-Mediated Uterine Peristalsis and Associated Embryo Implantation Impairments in Mouse Adenomyosis
Source: Front Physiol. 2021 Nov 4;12:744745. doi: 10.3389/fphys.2021.744745 (PMC8599363; doi:10.3389/fphys.2021.744745)

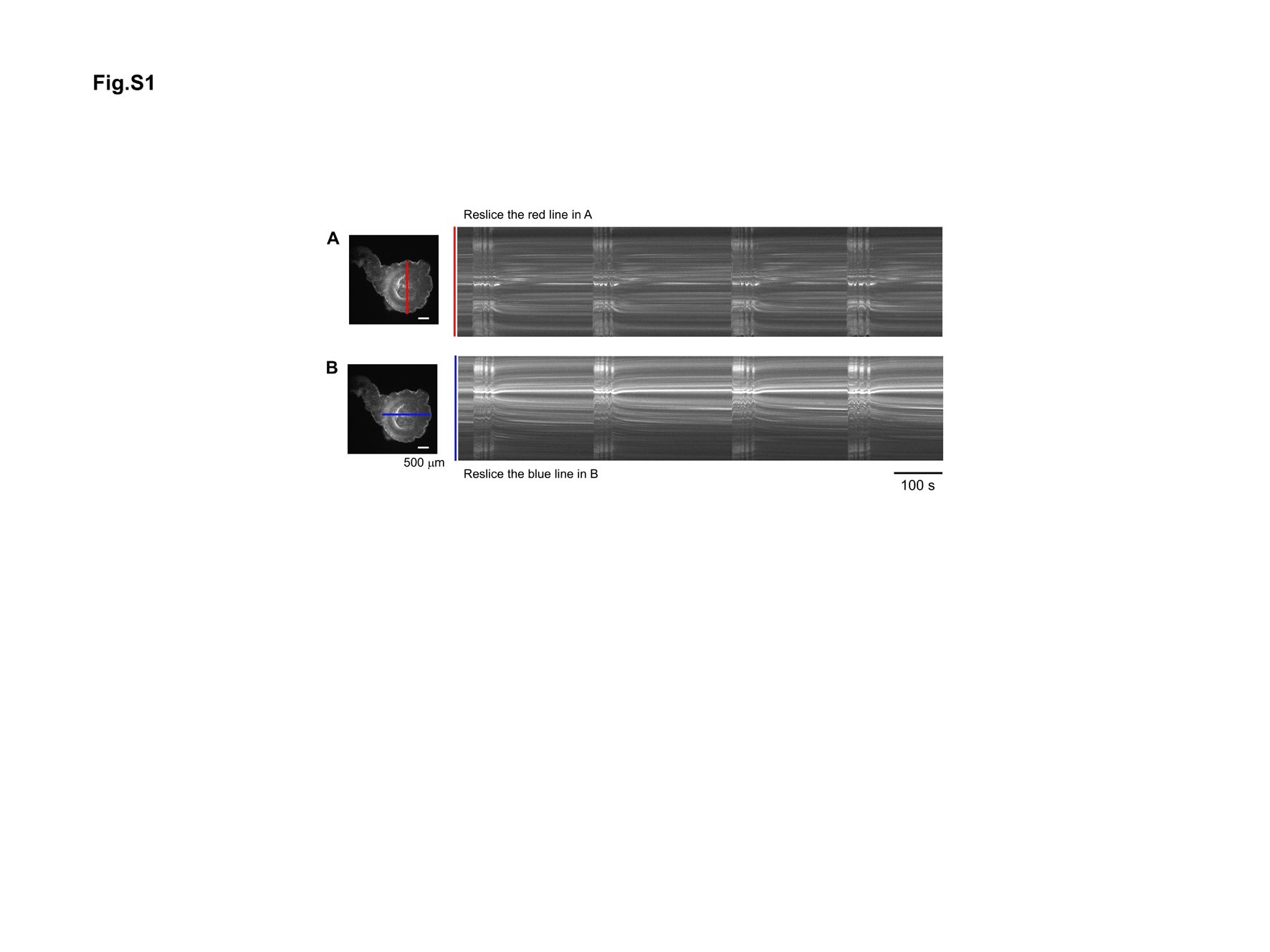

Supplement: Supplementary Figure 1 — Uterine peristalsis causes a repetitive spatio-temporal change in uterine slices as revealed by reslicing through the two marked lines to produce kymographs. [file Image_1.TIF]

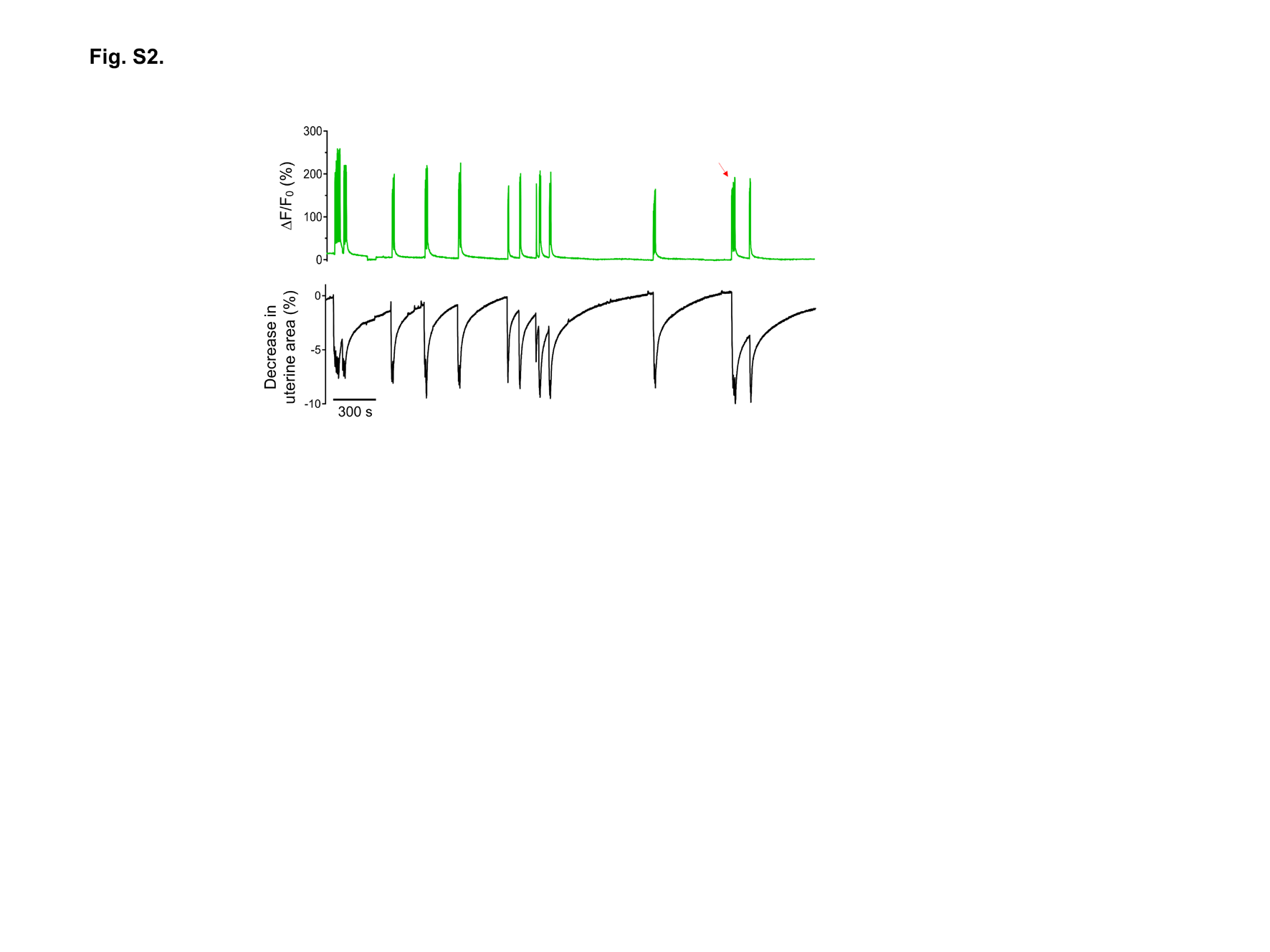

Supplement: Supplementary Figure 2 — Ca2+ signals as revealed by the R-CaMP1.07 Ca2+ reporter showing a close temporal association between Ca2+ oscillations and uterine peristalsis. R-CaMP1.07 fluorescence was recorded with a 4× objective (Nikon, Tokyo, Japan) at a speed of 3 Hz. The 568 nm line of an argon-ion laser provided fluorescence excitation, with a shutter to control exposure duration; emission of the Ca2+ indicator was monitored at wavelengths >600 nm. Changes in uterine area and fluorescence were calculated as described under the methods and materials section. [file Image_2.TIF]
